# Reply to Bronstein and Vinogradov

**DOI:** 10.15252/embr.202152500

**Published:** 2021-02-18

**Authors:** Emilia Niemiec

**Affiliations:** ^1^ Medical Ethics Division Department of Clinical Sciences Lund University Lund Sweden

**Keywords:** S&S: Economics & Business, S&S: Ethics

## Abstract

The response by the author.

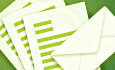

I thank Michael Bronstein and Sophia Vinogradov for their interest and comments. I would like to respond to a few of their points.

First, I agree with the authors that empirical studies should be conducted to validate any approaches to prevent the spread of misinformation before their implementation. Nonetheless, I think that the ideas I have proposed may be worth further discussion and inspire empirical studies to test their effectiveness.

Second, the authors warn that informing about the imperfections of scientific research may undermine trust in science and scientists, which could result in higher vulnerability to online health misinformation (Roozenbeek *et* *al*, 2020; Bronstein & Vinogradov, 2021). I believe that transparency about limitations and problems in research does not necessarily have to diminish trust in science and scientists. On the contrary, as Veit *et al* put it, “such honesty… is a prerequisite for maintaining a trusting relationship between medical institutions (and practitioners) and the public” (Veit *et* *al*, 2021). Importantly, to give an honest picture of scientific research, information about its limitations should be put in adequate context. In particular, the public also should be aware that “good science” is being done by many researchers; we do have solid evidence of effectiveness of many medical interventions; and efforts are being taken to address the problems related to quality of research.

Third, Bronstein and Vinogradov suggest that false and dangerous information should be censored. I agree with the authors that “[c]ensorship can prevent individuals from being exposed to false and potentially dangerous ideas” (Bronstein & Vinogradov, 2021). I also recognize that some information is false beyond any doubt and its spread may be harmful. What I am concerned about are, among others, the challenges related to defining what is dangerous and false information and limiting censorship only to this kind of information. For example, on what sources should decisions to censor be based and who should make such decisions? Anyone, whether an individual or an organization, with a responsibility to censor information will likely not only be prone to mistakes, but also to abuses of power to foster their interests. Do the benefits we want to achieve by censorship outweigh the potential risks?

Fourth, we need rigorous empirical studies examining the actual impact of medical misinformation. What exactly are the harms we try to protect against and what is their scale? This information is necessary to choose proportionte and effective measures to reduce the harms. Bronstein and Vinogradov give an example of a harm which may be caused by misinformation—an increase in methanol poisoning in Iran. Yet, as noticed by the authors, misinformation is not the sole factor in this case; there are also cultural and other contexts (Arasteh *et* *al*, 2020; Bronstein & Vinogradov, 2021). Importantly, the methods of studies exploring the effects of misinformation should be carefully elaborated, especially when study participants are asked to self‐report. A recent study suggests that some claims about the prevalence of dangerous behaviors, such as drinking bleach, which may have been caused by misinformation are largely exaggerated due to the presence of problematic respondents in surveys (preprint: Litman *et* *al*, 2021).

Last but not least, I would like to call attention to the importance of how veracity of information is determined in empirical studies on misinformation. For example, in a study of Roozenbeek *et al*, cited by Bronstein and Vinogradov, the World Health Organization (WHO) was used as reliable source of information, which raises questions. For instance, Roozenbeek *et* *al* (2020) used a statement “the coronavirus was bioengineered in a military lab in Wuhan” as an example of false information, relying on the judgment of the WHO found on its “mythbusters” website (Roozenbeek *et* *al*, 2020). Yet, is there a solid evidence to claim that this statement is false? At present, at least some scientists declare that we cannot rule out that the virus was genetically manipulated in a laboratory (Relman, 2020; Segreto & Deigin, 2020). Interestingly, the WHO also no longer excludes such a possibility and has launched an investigation on this issue (https://www.who.int/health‐topics/coronavirus/origins‐of‐the‐virus, https://www.who.int/emergencies/diseases/novel‐coronavirus‐2019/media‐resources/science‐in‐5/episode‐21‐‐‐covid‐19‐‐‐origins‐of‐the‐sars‐cov‐2‐virus); the information about the laboratory origin of the virus being false is no longer present on the WHO “mythbusters” website (https://www.who.int/emergencies/diseases/novel‐coronavirus‐2019/advice‐for‐public/myth‐busters). Against this backdrop, some results of the study by Roozenbeek *et* *al* (2020) seem misleading. In particular, the perception of the reliability of the statement about bioengineered virus by study participants in Roozenbeek *et* *al* (2020) does not reflect the susceptibility to misinformation, as intended by the researchers, but rather how the respondents perceive reliability of uncertain information.

I hope that discussion and research on these and related issues will continue.

## References

[embr202152500-bib-0001] Arasteh P , Pakfetrat M , Roozbeh J (2020) A surge in methanol poisoning amid COVID‐19 pandemic: why is this occurring? Am J Med Sci 360: 201 32536420 10.1016/j.amjms.2020.05.019 PMC7237356

[embr202152500-bib-0002] Bronstein MV , Vinogradov S (2021) EMBO Rep 22:e52282 10.15252/embr.202052282 PMC7926209 33599078

[embr202152500-bib-0003] Litman L , Rosen Z , Rosenzweig C , Weinberger‐Litman SL , Moss AJ , Robinson J (2021) Did people really drink bleach to prevent COVID‐19? A tale of problematic respondents and a guide for measuring rare events in survey data. *medRxiv* 10.1101/2020.12.11.20246694 [PREPRINT] PMC10321604 37406017

[embr202152500-bib-0004] Relman DA (2020) Opinion: to stop the next pandemic, we need to unravel the origins of COVID‐19. Proc Natl Acad Sci USA 117: 29246–29248 33144498 10.1073/pnas.2021133117 PMC7703598

[embr202152500-bib-0005] Roozenbeek J , Schneider CR , Dryhurst S , Kerr J , Freeman ALJ , Recchia G , van der Bles AM , van der Linden S (2020) Susceptibility to misinformation about COVID‐19 around the world. R Soc Open Sci 7: 201199 33204475 10.1098/rsos.201199 PMC7657933

[embr202152500-bib-0006] Segreto R , Deigin Y (2020) The genetic structure of SARS‐CoV‐2 does not rule out a laboratory origin: SARS‐COV‐2 chimeric structure and furin cleavage site might be the result of genetic manipulation. BioEssays 10.1002/bies.202000240 PMC7744920 33200842

[embr202152500-bib-0007] Veit W , Brown R , Earp BD (2021) In science we trust? Being honest about the limits of medical research during COVID‐19. Am J Bioeth 21: 22–24 33373581 10.1080/15265161.2020.1845861

